# Paranoid Ideation and Violence: Meta-analysis of Individual Subject Data of 7 Population Surveys

**DOI:** 10.1093/schbul/sbw006

**Published:** 2016-02-15

**Authors:** Jeremy W. Coid, Simone Ullrich, Paul Bebbington, Seena Fazel, Robert Keers

**Affiliations:** ^1^Violence Prevention Research Unit, Wolfson Institute of Preventive Medicine, Queen Mary University of London, London, UK;; ^2^Division of Psychiatry, Faculty of Brain Sciences, University College London, London, UK;; ^3^Department of Psychiatry, University of Oxford, Oxford, UK;; ^4^Department of Biological and Experimental Psychology, School of Biological and Chemical Sciences, Queen Mary University of London, London, UK

**Keywords:** psychotic-like-experiences, severity and victims of violence, psychiatric comorbidity

## Abstract

There is controversy whether associations between psychosis and violence are due to coexisting substance misuse and factors increasing risk in nonpsychotic persons. Recent studies in clinical samples have implicated independent effects of paranoid delusions. Research findings suggest that individual psychotic-like-experiences on the psychosis continuum in the general population are associated with violence; it remains unclear whether this association is due to psychiatric comorbidity. We pooled data from 7 UK general population surveys (*n* = 23 444) and conducted a meta-analysis of individual subject data. Further meta-analyses were performed to identify heterogeneity. Main exposure variables: 5 psychotic-like-experiences and a categorical measure of psychosis. Comorbidity was established through standardized self-report instruments. Information was collected on violence, severity, victims. Paranoid ideation was associated with violence (AOR 2.26, 95% CI 1.75–2.91), severity and frequency, even when controlling for effects of other psychotic-like-experiences. Associations were not explained by comorbid conditions, including substance dependence. Psychotic disorder was associated with violence and injury to the perpetrator but associations were explained by paranoid ideation. Individual associations between hypomania, thought insertion, hallucinations, and violence were nonsignificant after adjustments, and significantly associated only when comorbid with antisocial personality disorder. Strange experiences were only associated with intimate partner violence. Paranoid ideation on a psychosis-continuum in the general population was associated with violence. All other associations were explained by comorbidity. Further investigation should determine whether paranoid ideation among persons in the community require preventive interventions, similar to those presenting to mental health services. Nevertheless, risks are considerably increased for psychotic-like-experiences with co-occurring antisocial personality disorder.

## Introduction

Psychotic-like-experiences (PLEs) are relatively common in the general population and on a continuum with psychotic symptoms seen in clinical samples.^1^ The demographic and comorbid risk factors associated with PLEs in the general population^2^ correspond to those for violence.^3^ However, findings of associations between individual PLEs and violence at the population level are inconsistent. Four community surveys demonstrated that paranoid ideation and delusions of threat or persecution are associated with violence,^4–7^ but findings regarding hallucinations, thought interference, and external influences or control were conflicting.

The majority of studies of clinical samples have investigated associations with clinical diagnoses, and these findings have also been inconsistent.^8^ It has been argued that risk factors for violence among the mentally ill are the same as those for nonpsychotic persons.^9^ Reanalysis of these data, however, has demonstrated that neglecting spatio-temporal contiguity of exposure and outcome leads to an underestimation of the strength of association. When temporal proximity of mental illness and violence (within 12 months) was considered, a statistically significant, yet modest relationship was found.^10^ A large-scale epidemiological study investigating the association with violence in schizophrenia patients living in the community identified distinct but overlapping risk factors for minor and serious violence including positive psychotic symptoms, co-occurring substance abuse, interpersonal and social factors, childhood conduct problems and victimization.^11^ More recently, meta-analyses and case register studies concluded that psychiatric disorders are associated with violence, but that the relationship is largely or entirely explained by comorbid substance misuse.^12–14^

These inconsistencies are likely to be explained by the considerable methodological heterogeneity of studies included in previous meta-analyses.^8^ Meta-analysis of individual participant data are therefore preferable to standard meta-analyses, particularly when there are few relevant studies and subgroup analyses are required. Accordingly, we carried out a combined analysis of 7 community surveys of violence and PLEs using representative samples from England, Scotland, and Wales. The same instruments were administered in each survey to measure PLEs and violence. Our aims were to investigate: (1) which experiences showed strongest associations with self-reported violence, (2) whether associations with psychotic disorder and PLEs differed according to severity and victim type, and (3) the effects of psychiatric comorbidity on these associations.

## Methods

### Sample

Data from 7 community surveys of psychiatric morbidity were included: the 2000 household survey of Great Britain (*n* = 8580),^15^ the 2007 household survey of England (*n* = 7403),^16^ and the second Men’s Modern Lifestyles Survey (MMLS), which included 18–34 year old men.^17^ The latter is a nationally representative sample (*n* = 3247), with 4 additional boost survey samples of Black and minority ethnic men (*n* = 1540), men from low socioeconomic background or unemployed (*n* = 1002), men from the London borough of Hackney (*n* = 883), and men from Glasgow East, Scotland (*n* = 789).

Survey details have been described elsewhere.^15–17^ In brief, computer-assisted interviews with men and women age 16+ were carried out in the Household surveys. The “small users” Postcode Address File is a comprehensive list of addresses at which mail may be delivered. It was created by the UK post office and is hierarchically organized on a geographic basis. Postcodes may have up to 7 characters and correspond closely to electoral wards. Lowest level units are known as “delivery points” of which there are approximately 17 per postcode. It covers addresses which receive less than 25 items of mail per day and was used as the household sampling frame. The Kish Grid method^18^ was applied to select 1 person from each household.

The MMLS used random location methodology, an advanced form of quota sampling shown to reduce biases introduced when interviewers choose locations to sample. Participants completed a self-administered pencil and paper questionnaire in private.

### Measures

Demographic characteristics were gathered via self-report and the study participants ascribed themselves to 9 ethnic groups. Social class was assessed using the Standard Occupational Classification 1991,^19^ an ordinal classification system: (1) professional occupations; (2) managerial and technical occupations; (3) skilled occupations, nonmanual and manual; (4) partly skilled occupations; and (5) unskilled occupations. Each survey assessed psychopathology and violent behavior using standardized self-report instruments. Items of the Psychosis Screening Questionnaire (PSQ)^20^ cover hypomania, thought interference, paranoid ideation, strange experiences, and auditory and visual hallucinations experienced in the past year. A score of 3+ symptoms was applied to identify probable psychotic disorder. This gave a general population prevalence only slightly lower than that commonly found for psychotic illness which is estimated to range between 1.3% and 2.2%.^21^ Alcohol dependence was identified by a score of 20+ on the Alcohol Use Disorders Identification Test (past year).^22^ We administered the Structured Clinical Interview for DSM-IV personality disorders—screening questionnaire (SCID-II screen)^23^ to identify antisocial personality disorder (ASPD) which reflects a combination of conduct disorder (before the age of 15 years) and the adult antisocial syndrome (persistent antisocial behaviors since age 18).

The surveys differed only in the instruments used to measure depression, anxiety disorder, and drug dependence. In the MMLS, the Hospital Anxiety and Depression Scale^24^ was used to rate anxiety disorder and depression, based on a score of 11+ (on depressive and anxiety symptoms, respectively) in the past week. In the household surveys, the Revised Clinical Interview Schedule (CISR)^25^ provided ratings of 6 categories of anxiety disorder over the same time period which were then combined to a diagnosis of “any anxiety disorder” and 1 rating of “depressive episode” (past week). Drug dependence (past year) was identified by the Drug Use Disorders Identification Test (DUDIT)^26^ at a score of 25+ in the MMLS, while 5 questions applied to reported use of different substances captured drug dependence in the Household surveys.^15,16^

In all surveys a history of violence was established by asking participants: “Have you been in a physical fight, assaulted or deliberately hit anyone in the past five years?” Additional questions covered characteristics, severity and victims, whereby 12 specific outcomes were identified: repetitive violence (at least 5 incidents over the previous 5 years); violence when intoxicated (perpetrator was intoxicated); victim versatility (at least 3 different types of victim); incidents in which the victim was injured; incidents in which the perpetrator was injured; incidents involving the police, minor violence (where neither victim nor perpetrator were injured and police was not involved); intimate partner violence (where spouses/ cohabiting partners and girlfriends/ boyfriends were victims); violence toward other family members including children; violence toward a friend; violence toward other acquaintances; and violence toward a stranger.

### Statistical Analysis

We conducted an aggregate meta-analysis of individual-level data in the 7 samples. This provided maximum statistical power by combining studies that varied in size, and, in contrast to conventional meta-analyses, can incorporate individual-level confounders. The standard approach to meta-analysis of individual subject data is mixed effect-modeling where heterogeneity in the pooled samples is accounted for by modeling random effects of the samples and fixed effects of covariates. As can be seen in supplementary table 1, the 7 pooled samples under study differed significantly in all exposure and outcome variables. To control for differences between samples and to account for correlations within surveys, we included survey type as a random effect.

We first identified potential confounders for violence by comparing demographic and psychopathological attributes of violent and nonviolent participants using logistic mixed models. Factors were included separately in unadjusted analyses, and simultaneously in adjusted analyses (table 1). In all subsequent analyses we adjusted for demographic (gender, age, marital status, social class, ethnicity) and clinical characteristics (alcohol/ drug dependence, ASPD, depression, anxiety disorder) significantly associated with violent outcome (confounders).

**Table 1.** Associations Between Demographic and Clinical Characteristics and Violence

	No Violence	Violence	Adjusted Only for the Random Effects of Sample			Simultaneous Inclusion of All Variables		
	*N* (%)	*N* (%)	OR	95% CI	*P*	OR	95% CI	*P*
Gender								
Female	8413 (95.4)	404 (4.6)	Ref.					
Male	11 413 (80.9)	2697 (19.1)	2.96	2.62–3.35	<.001	2.95	2.56–3.41	<.001
Age								
16–34	6866 (74.3)	2373 (25.7)	Ref.					
35–54	6706 (91.6)	614 (8.4)	0.33	0.30–0.37	<.001	0.38	0.34–0.43	<.001
55+	6225 (98.4)	104 (1.6)	0.07	0.06–0.08	<.001	0.09	0.07–0.11	<.001
Marital status								
Married	11 317 (92.3)	940 (7.7)	Ref.					
Single	5566 (75.2)	1836 (24.8)	2.99	2.73–3.27	<.001	1.49	1.33–1.66	<.001
Separated/ divorced	2887 (90.2)	315 (9.8)	1.43	1.25–1.64	<.001	1.58	1.34–1.86	<.001
Social class								
I–II	5936 (93.0)	448 (7.0)	Ref.					
III–V/ not classified	12 448 (84.6)	2267 (15.4)	1.76	1.57–1.96	<.001	1.64	1.44–1.85	<.001
Employment								
Employed	18 270 (88.3)	2424 (11.7)	Ref.					
Unemployed	1341 (69.0)	603 (31.0)	1.79	1.59–2.01	<.001	1.07	0.93–1.24	.316
Ethnicity								
White	16 789 (87.0)	2509 (13.0)	Ref.					
Black Caribbean	350 (79.9)	88 (20.1)	1.33	1.02–1.72	.030	1.05	0.76–1.45	.728
Black African	395 (80.8)	94 (19.2)	1.00	0.77–1.29	.981	0.65	0.47–0.92	.015
Black other	34 (72.3)	13 (27.7)	1.82	0.93–3.56	.078	1.51	0.69–3.27	.292
Indian	569 (89.3)	68 (10.7)	0.55	0.41–0.73	<.001	0.41	0.28–0.59	<.001
Pakistani	588 (84.9)	105 (15.1)	0.72	0.55–0.92	.011	0.57	0.42–0.78	.001
Bangladeshi	189 (90.0)	21 (10.0)	0.42	0.26–0.68	<.001	0.36	0.20–0.65	.001
Chinese	90 (81.1)	21 (18.9)	1.12	0.68–1.85	.643	0.92	0.42–1.99	.843
Other	745 (81.3)	171 (18.7)	1.02	0.83–1.25	.826	0.80	0.62–1.02	.076
Alcohol dependence								
No	18 714 (88.6)	2402 (11.4)	Ref.					
Yes	936 (59.5)	636 (40.5)	5.12	4.55–5.75	<.001	2.09	1.80–2.43	<.001
Drug dependence								
No	19 303 (87.8)	2694 (12.3)	Ref.					
Yes	290 (44.7)	359 (55.3)	11.1	9.35–13.18	<.001	2.17	1.75–2.71	<.001
ASPD								
No	19 315 (89.7)	2226 (10.3)	Ref.					
Yes	428 (34.0)	832 (66.0)	12.94	11.36–14.73	<.001	6.57	5.35–7.21	<.001
Depressive disorder								
No	18 744 (86.9)	2820 (13.1)	Ref.					
Yes	980 (79.2)	258 (20.8)	1.18	1.01–1.37	.034	0.79	0.68–1.02	.020
Anxiety disorder								
No	16 984 (88.2)	2283 (11.9)	Ref.					
Yes	2752 (77.6)	794 (22.4)	2.41	2.19–2.65	<.001	1.83	1.59–2.06	<.001

*Note*: ASPD, antisocial personality disorder.

We performed logistic mixed models to test for associations between psychotic disorder and violent outcomes. We utilized a similar approach to test for associations with each of the 5 PLEs, incorporating all 5 experiences simultaneously to ascertain their independent effects. We then tested the effect of a combination of hallucinations and persecutory ideation to test the effect on violent outcomes. In order to control for effects of multiple testing, findings were only considered statistically significant at the Bonferroni corrected significance threshold of *P* < .003 (0.05/13).

Individual-level analysis allowed us to control for effects of survey type on outcome and of confounders that differed between studies. However, we could not exclude the possibility of unmeasured heterogeneity between samples. We therefore conducted a meta-analysis with random effects to confirm the findings.

In order to investigate the impact of co-occurring psychiatric disorders further, we tested associations between a categorical diagnosis of putative psychotic disorder and each PLE with violence in individuals with and without comorbid depression, anxiety, ASPD, alcohol dependence, and drug dependence.

## Results

Violence in the past 5 years was reported by 3101 (13.5%) of a combined sample of 23 444 survey respondents. At least 1 PLE was reported by 1780 participants (7.7 %): 300 (1.3%) reported hypomania, 380 (1.6%) thought insertion, 641 (2.8%) paranoid ideation, 1022 (4.4%) strange experiences, and 360 (1.6%) hallucinations. In total, 220 participants (1.0%) reported 3 or more symptoms to screen positive for our categorical measure of putative psychotic disorder. Only 2 among those did not report any comorbid psychopathology.

Of those with putative psychotic disorder, 116 (53.7%) reported having used mental health care services. At the individual PLE level, 84 people with hypomania (28.5%), 126 of those with thought insertion (34.3%), 228 of those with paranoid ideation (36.0%), 311 of those reporting strange experiences (30.9%), and 153 with hallucinations (43.5%) had been in contact with mental health services.

Of people with a putative psychotic disorder, 110 (52.6%) reported violent behavior in the past 5 years. At the level of individual PLEs, violence was reported by 122 (42.1%) of those with hypomania, 129 (34.9%) of those with thought insertion, 322 (52.0%) of those with paranoid ideation, 358 (36.2%) of those reporting strange experiences, and 138 (40.0%) of those with hallucinations.

Associations between demographic characteristics, psychopathology, and violence are reported in table 1. Significant demographic risk factors for violence in adjusted analyses included being young, male, unemployed, single, separated or divorced, and low socioeconomic status. Following adjustment, being of Black African, Indian, Pakistani, and Bangladeshi ethnic origin conveyed protective effects for the perpetration of violence compared to White study participants. The increased risk of violence in Black Caribbean study participants was no longer significant after simultaneous inclusion of all demographic and clinical variables. The presence of ASPD, anxiety, depression and alcohol and drug dependence significantly increased the risk of violence. After adjustment, however, depression was no longer a risk factor for violence but conveyed protective effects.

### Psychotic–Like Experiences, Psychotic Disorder, and Violence

Following adjustments for the demographic and clinical characteristics described above, our measure of psychotic disorder was significantly associated with several violent outcomes. Two out of 13 of these associations—violence of any kind and injury to the perpetrator—remained significant after correction for multiple testing (table 2). Findings were similar for the individual PLEs of hypomania, thought insertion, strange experiences, and hallucinations. Few specific associations were observed, and none remained after correction for multiple testing apart from the relationship between strange experiences and intimate partner violence (table 2). Furthermore, none of the associations with a combined measure of paranoid ideation and hallucinations were statistically significant following correction for multiple testing.

**Table 2.** Associations Between Psychosis, Psychotic Symptoms, and Violence

	PSQ 3+	Hypomania	Thought Insertion	Paranoid Ideation	Strange Experiences	Hallucinations
	OR (95% CI)^a^	OR (95% CI)^b^	OR (95% CI)^b^	OR (95% CI)^b^	OR (95% CI)^b^	OR (95% CI)^b^
Any violence	1.97 (1.32–2.93)*	1.38 (0.96–1.99)	1.10 (0.78–1.56)	2.26 (1.75–2.91)*	1.07 (0.85–1.34)	1.26 (0.88–1.80)
Repetitive violence	1.98 (1.19–3.31)	1.67 (0.98–2.85)	0.65 (0.37–1.16)	2.57 (1.81–3.66)*	0.93 (0.64–1.36)	1.67 (0.99–2.81)
When intoxicated	1.43 (0.90–2.28)	0.89 (0.56–1.43)	1.00 (0.64–1.57)	1.74 (1.28–2.35)*	1.02 (0.76–1.37)	1.24 (0.80–1.91)
Severity of violence						
Victim versatility	1.55 (0.86–2.77)	0.98 (0.49–1.94)	1.09 (0.57–2.09)	1.41 (0.92–2.16)	1.18 (0.76–1.84)	2.02 (1.14–3.58)
Victim injured	0.91 (0.57–1.45)	1.45 (0.93–2.26)	0.46 (0.28–0.77)	1.78 (1.33–2.40)*	1.11 (0.82–1.49)	0.87 (0.55–1.38)
Perpetrator injured	2.10 (1.38–3.19)*	1.45 (0.95–2.22)	0.91 (0.60–1.40)	1.65 (1.23–2.19)*	1.03 (0.78–1.38)	1.66 (1.11–2.49)
Police involved	1.76 (1.12–2.75)	1.50 (0.95–2.37)	0.95 (0.60–1.50)	1.93 (1.43–2.60)*	1.35 (1.00–1.82)	1.26 (0.81–1.95)
Minor violence	0.97 (0.55–1.71)	0.68 (0.37–1.25)	1.34 (0.82–2.19)	0.92 (0.62–1.35)	0.86 (0.60–1.23)	1.09 (0.64–1.84)
Victim of violence						
Intimate partner	1.79 (1.11–2.88)	1.32 (0.78–2.25)	1.23 (0.76–2.00)	1.69 (1.19–2.29)	1.69 (1.22–2.35)*	0.71 (0.43–1.18)
Family member	2.04 (1.21–3.45)	1.23 (0.67–2.26)	1.35 (0.76–2.42)	1.75 (1.16–2.63)	0.97 (0.63–1.51)	1.57 (0.90–2.73)
Friend	1.17 (0.71–1.92)	1.52 (0.91–2.82)	0.81 (0.47–1.38)	1.27 (0.89–1.80)	0.92 (0.64–1.33)	1.29 (0.77–2.15)
Person known	0.58 (0.33–1.02)	1.04 (0.62–1.76)	0.77 (0.45–1.31)	1.20 (0.87–1.67)	1.03 (0.75–1.41)	0.90 (0.55–1.49)
Stranger	1.69 (1.11–2.57)	0.97 (0.63–1.50)	1.03 (0.68–1.57)	1.92 (1.45–2.54)*	0.99 (0.74–1.31)	1.41 (0.94–2.11)

*Note*: PSQ, Psychosis Screening Questionnaire.

^a^Adjusted for random effects of sample and fixed effects of gender, age, marital status, employment, social class, ethnicity and comorbid drug and alcohol dependence, depression, anxiety, and ASPD.

^b^Further adjusted for occurrence of other psychotic symptoms.

*Remained significant following correction for multiple testing (*P* < .003).

In contrast, after correction for multiple testing and following adjustment for demography and clinical characteristics, paranoid ideation remained associated with violence of any kind, repetitive violence, violence when intoxicated, violence resulting in injury to perpetrator or victim, police involvement, and violence against strangers.

**Fig. 1.**
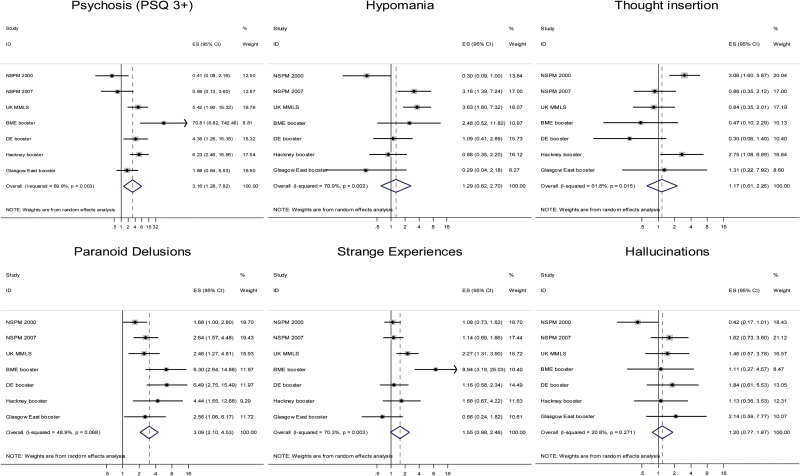
Forest plots from meta-analyses of psychosis (Psychosis Screening Questionnaire [PSQ 3+]) and psychotic-like-experiences (PLEs) on any violence.

Figure 1 shows results of the meta-analysis, confirming that putative psychosis and paranoid ideation were associated with all types of violence in each of the 7 surveys. Findings related to hypomania, thought insertion, and strange experiences corresponded to those from the meta-analysis of individual subject data, in showing no significant associations. Although analyses in the case of putative psychosis, hypomania, thought insertion, and strange experiences showed significant heterogeneity, none was observed for either paranoid ideation or hallucinations.

### Gender Specific Associations

Gender specific associations were tested by including an interaction term in the statistical model. The category of psychosis demonstrated no significant gender interactions with any violent outcomes following adjustment for demography and comorbidity, and after correction for multiple testing.

Subsequent testing of the individual PLEs showed a significant gender-interaction term, thought insertion and police involvement in the violent incident (AOR 0.18, 95% CI 0.07–0.46, *P* < .001) indicative of the effects being statistically stronger in women. No other gender specific pattern was apparent.

### Effects of Comorbid Disorders

**Fig. 2.**
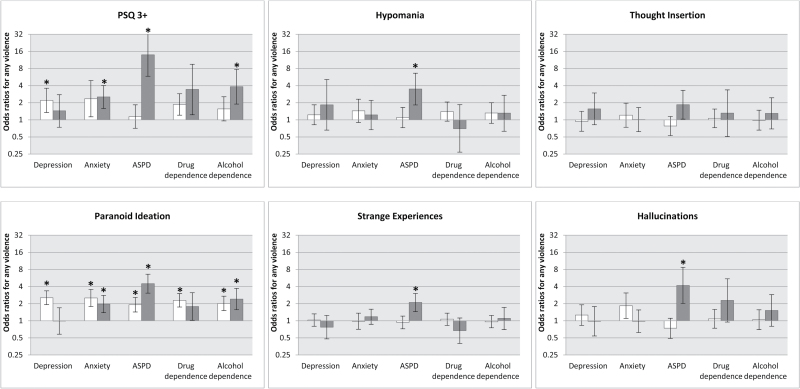
Associations with violence on diagnosis/ symptom level in the absence and presence of comorbid mental disorders. White bars reflect the absence, black bars the presence of comorbidity. *Remained significant following correction for multiple testing (*P* < .003).

Figure 2 shows the effects of comorbid psychiatric disorders on associations of putative psychosis and each PLE with violence. The association of putative psychosis with violence was wholly due to comorbidity, being significant only among individuals with comorbid anxiety, ASPD, or alcohol dependence. Only in the absence of depression was psychotic disorder significantly associated with violence.

The remaining panels in figure 2 show that hypomania, strange experiences, and hallucinations were only associated with violence when comorbid with ASPD.

In contrast, comorbidity had no effect on the significant link between paranoid ideation and violence, with the exception that paranoid ideation was not significantly associated with violence when comorbid with depression and in the presence of drug dependence.

## Discussion

Our findings confirmed robust associations between paranoid ideation and violence in the UK adult general population. This relationship was not explained by comorbid substance abuse or other psychiatric comorbidity, contrasting with previous findings for psychotic disorder.^12–14^ Paranoid ideation was specifically associated with more serious violent incidents in which injuries were inflicted, repetitive violence, and incidents involving police; victims were more likely to be strangers. These associations were not the result of confounding by demographic factors or comorbid psychopathology, and were independent of other co-occurring psychotic symptoms. In contrast, hypomania, thought insertion, strange experiences and hallucinations were largely unrelated to violence.

In contrast to our findings for paranoid ideation, psychotic disorder was not independently associated with violence, confirming meta-analytic and case register studies.^12–14^ The association was only significant in the absence of depression and this reflected the effects of paranoid ideation. All other associations between violence and psychosis were specifically explained by comorbid anxiety disorder, alcohol dependence, or ASPD. Historically, it was thought that anxiety limited criminal and violent activity but more recent evidence suggests that heightened responsiveness to threat may lead to persistent violent behavior, a notion which corresponds to our findings.^27,28^

Contrary to previous research,^29,30^ associations of psychotic disorder and PLEs with violent outcome were not moderated by gender.

The prevalence of violence in the sub-sample with putative psychotic disorder was higher than reported in other epidemiological studies.^11^ However, a substantial proportion of the pooled surveys were young, single men, all risk factors for violent behavior which increased the prevalence of violent behavior.

### Delusions and Violence

Our findings are in line with previous studies showing that, in a range of psychotic symptoms, persecutory ideation is most strongly associated with violence.^4–7^ Early studies suggested that violence can be driven by psychotic symptoms, particularly delusions,^5,31–33^ but subsequent research failed to replicate these findings,^34–36^ or only partially supported them.^37,38^ Our findings, therefore, correspond to more recent studies in demonstrating relationships between delusions and violence similar to those found in clinical samples.^39,40^

Apart from paranoid ideation, associations between PLEs and violence in this study were entirely explained by confounding by other symptoms and comorbid psychopathology. A higher risk of violent convictions has been reported among released prisoners and patients with bipolar disorder, but was considered the result of comorbid substance misuse.^14^ Previous population studies have reported inconsistent findings for hallucinations.^6,7,30^ This could be due to differences in measurement both of PLEs and violence. However, our findings also indicate that the relationship is explained by comorbid psychopathology. Although hallucinations and delusions typically occur together in psychosis and are considered risk factors for poor clinical outcome^41–43^ this combination was not associated with any violent outcome measure, and persecutory ideation constituted the main explanatory factor in the association with violence.

### Comorbidity in the Association With Violence

We confirmed previous findings that comorbid psychopathology is key to explaining the pathway between our categorical measure of psychosis and violence. However, in the general population this association was primarily explained by ASPD, to a lesser extent by substance misuse, and largely accounted for by alcohol rather than drug dependence. This may be explained by confounding effects of ASPD, which commonly coexists with substance misuse over the lifetime. Few previous studies have measured confounding from ASPD, and the effects of comorbid substance abuse in individuals with psychosis may therefore have been over-estimated.

### Limitations

Our study had several limitations. Violent behavior within the last 5 years was assessed via self-report. This may underestimate true prevalence, as socially undesirable behaviors tend to be less frequently reported. However, criminological studies have demonstrated a high validity of self-report of offending especially for men, White and Black study participants. The validity of self-report was lowest for Asian females.^44^ Diagnoses of Axis I and Axis II mental disorders were also derived from self-report questionnaires. A previous study investigating anxiety disorders has shown that self-report can compare favorably with clinician assessments.^45^ The highest odds ratios were achieved when investigating the association of a putative diagnosis of psychosis comorbid with ASPD. However, ASPD was diagnosed using criteria provided by DSM-IV. This set of criteria contains indicators of violent and antisocial conduct. It is therefore likely that the association between ASPD and violence was over-estimated due to overlap of exposure and outcome and should be interpreted with caution.

Although we used a screening instrument assessing only 5 PLEs, these are central to the diagnosis of psychosis, have good face validity, and are likely to be strongly associated with other symptoms in the syndrome of psychosis. The prevalence of our categorical measure of putative psychotic disorder was slightly lower than prevalence rates generally established through formal clinical evaluation,^21^ albeit of the right magnitude. Finally, although measures of psychosis and outcome were the same in all surveys, different measures of anxiety, depression and drug dependence were used, which may have resulted in some variation between samples. Furthermore, at first glance, our findings appear to contradict a recent study reporting depression as a risk factor for violence.^46^ Diagnoses in case register studies, however, are based on clinical judgment and are less inclusive with regard to comorbid mental disorders. Utilization of standardized diagnostic instruments (as in our study) allows adjusting for comorbidity and only after this adjustment depression was inversely associated with violent outcome. This finding could reflect the heterogeneity of depressive disorder where only a subgroup is at risk for violent behavior.

Modeling of interaction terms with gender did not show any gender moderated associations between PLEs and violence. However, women were underrepresented in these pooled analyses (women only participated in the 2 household surveys) and we may, therefore, have underestimated such effects.

To maximize statistical power, we pooled data from several population-based studies using the same instruments but conducted in different regions in England, Wales, and Scotland. While individual level analyses allowed us to control for the effects of survey type on outcome and for confounders that differed between the studies, we cannot rule out unmeasured heterogeneity between samples. Nevertheless, our major findings were confirmed by conventional meta-analysis using random effect models. There was however some heterogeneity as indicated by significant findings in *I* ^2^ tests for putative psychosis, hypomania, thought insertion, and strange experiences. This may reflect different prevalences of comorbid psychopathology and requires further study.

Finally, our study relied on data from cross-sectional surveys and inferences of causal relationships between psychosis and violence are thereby constrained. Population-based longitudinal surveys taking into consideration temporal proximity would increase plausibility. However, distinguishing between symptoms and diagnosis is critical to clarifying the association between mental illness and violence and has major implication for the management of those at risk.

### Implications

Evidence of a psychosis continuum in the general population^1^ is supported by observations that demography, nongenetic and genetic risk factors, and neurocognitive deficits apply similarly to the subclinical population as to those with clinical psychosis.^41^ Our findings provide new supporting evidence of the continuum by showing that patterns of violence associated with persecutory ideation also occur across the continuum. Epidemiological studies have shown that, whilst a small proportion of persons with subclinical psychotic symptoms go on to develop a clinical psychotic disorder, most are self-limiting and have good outcome.^47^ Nevertheless, our findings have implications for prevention and management of violence at the population level by demonstrating the key importance of paranoid ideation. These correspond to findings in clinical samples.^39,40^ Studies investigating clinical diagnoses are unable to detect associations at the symptom level and may thus lead to erroneous conclusions. The effects of paranoid ideation were direct, not explained by comorbid substance abuse or other comorbid psychopathology. However, negative affect, specifically anger due to delusional beliefs implying threat, is an important consideration in the pathway toward serious violence,^39,40^ together with increased risk conveyed by the emergence of persecutory delusions in those with psychotic illness who do not receive treatment.^48^ Providing treatment (including interventions for comorbid alcohol dependence), ensuring compliance, and monitoring people with a history of violence and persecutory delusions is indicated to prevent further violence in those with clinical psychosis. However, the most important area for future investigation is the prevention of violence at the population level from persons with paranoid ideation who are not in contact with healthcare services.

When communicating findings on the association between severe mental illness and violence it is important to note that psychotic illness/ symptoms of psychosis are one of many risk factors in the pathway toward violent behavior. Furthermore, the proportion of societal violence attributable to individuals with psychosis is relatively small,^49^ although higher in developing countries,^50^ and persons suffering from schizophrenia are approximately 14 times more likely to be victims of violence than to commit violence toward others.^51^

## Supplementary Material

Supplementary material is available at http://schizophreniabulletin.oxfordjournals.org.

## Funding

This study was funded by the UK National Institute for Health Research (NIHR) under its Program Grants for Applied Research funding scheme (RP-PG-0407-10500). The views expressed in this manuscript are those of the authors and not necessarily those of the UK National Health Service (NHS), the NIHR or the UK Department of Health. There was no editorial direction or censorship from the funders. S.F. was funded by a Wellcome Trust Senior Research Fellowship in Clinical Science (095806).

## Supplementary Material

Supplementary Data
