# Supplementary material for: Paranoid Ideation and Violence: Meta-analysis of Individual Subject Data of 7 Population Surveys
Source: Schizophr Bull. 2016 Feb 15;42(4):907–15. doi: 10.1093/schbul/sbw006 (PMC4903063; doi:10.1093/schbul/sbw006)
Supplement: Supplementary Data [file supp_42_4_907__index.html]

Paranoid Ideation and Violence: Meta-analysis of Individual Subject Data of 7 Population Surveys — Paranoid Ideation and Violence: Meta-analysis of Individual Subject Data of 7 Population Surveys — Supplementary Data 

# Paranoid Ideation and Violence: Meta-analysis of Individual Subject Data of 7 Population Surveys

## Supplementary Data

Data files

- Supplementary Data - Supplementary Data
