# Supplementary material for: Paranoid Ideation and Violence: Meta-analysis of Individual Subject Data of 7 Population Surveys
Source: Schizophr Bull. 2016 Feb 15;42(4):907–15. doi: 10.1093/schbul/sbw006 (PMC4903063; doi:10.1093/schbul/sbw006)
Supplement: Supplementary Data [file supp_sbw006_Supplemental_Table_1.docx]

**Supplemental Table 1.** A comparison of the seven population surveys

|  | Survey 1 | Survey 2 | Survey 3 | Survey 4 | Survey 5 | Survey 6 | Survey 7 |  |
| --- | --- | --- | --- | --- | --- | --- | --- | --- |
|  | n (%) | n (%) | n (%) | n (%) | n (%) | n (%) | n (%) | P^1)^ |
| **Demography** |  |  |  |  |  |  |  |  |
| Gender |  |  |  |  |  |  |  | <0.001 |
| Male | 3,852 (44.9) | 3,197 (43.2) | 1,540 (100.0) | 1,002 (100.0) | 789 (100.0) | 883 (100.0) | 3,247 (100.0) |  |
| Female | 4,728 (55.1) | 4,206 (56.8) | --- | --- | --- | --- | --- |  |
| Age |  |  |  |  |  |  |  | <0.001 |
| 16-34 | 2,477 (28.9) | 1,603 (21.7) | 1,098 (71.5) | 654 (66.1) | 780 (99.1) | 822 (93.1) | 2,046 (63.6) |  |
| 35-54 | 3,393 (39.6) | 2,543 (34.4) | 351 (22.9) | 251 (25.4) | 7 (0.9) | 59 (6.7) | 850 (26.4) |  |
| 55+ | 2,710 (31.6) | 3,257 (44.0) | 86 (5.6) | 85 (8.6) | --- | 2 (0.23) | 323 (10.0) |  |
| Marital status |  |  |  |  |  |  |  | <0.001 |
| Married | 4,383 (51.1) | 5,082 (68.7) | 611 (40.6) | 420 (42.1) | 192 (24.4) | 305 (34.7) | 1,589 (46.3) |  |
| Single | 2,279 (26.6) | 1,428 (19.3) | 836 (55.5) | 480 (48.1) | 550 (70.0) | 518 (59.0) | 1,512 (47.0) |  |
| Separated/ divorced | 1,918 (22.4) | 893 (12.1) | 59 (3.9) | 97 (9.7) | 44 (5.6) | 55 (6.3) | 216 (6.7) |  |
| Social class III-V | 5,414 (65.5) | 4,380 (62.4) | 968 (82.9) | 848 (97.9) | 655 (94.0) | 583 (76.9) | 2,195 (80.7) | <0.001 |
| Unemployed | 260 (3.1) | 164 (2.2) | 251 (16.7) | 354 (37.4) | 318 (41.8) | 133 (15.9) | 557 (17.9) | <0.001 |
| Ethnicity |  |  |  |  |  |  |  | <0.001 |
| White | 8031 (94.3) | 6807 (92.6) | --- | 842 (84.6) | 776 (98.7) | 284 (32.3) | 2901 (89.6) |  |
| Black Caribbean | 117 (1.4) | 104 (1.41) | 106 (6.9) | 23 (2.3) | --- | 74 (8.4) | 36 (1.1) |  |
| Black African | 54 (0.6) | 78 (1.1) | 175 (11.4) | 31 (3.1) | 3 (0.4) | 112 (12.7) | 52 (1.6) |  |
| Black other | 14 (0.2) | 6 (0.1) | 7 (0.5) | 2 (0.2) | --- | 14 (1.6) | 4 (0.1) |  |
| Indian | 82 (1.0) | 82 (1.7) | 298 (19.4) | 15 (1.5) | --- | 69 (7.8) | 69 (2.1) |  |
| Pakistani | 49 (0.6) | 49 (0.7) | 442 (28.8) | 22 (2.2) | 3 (0.4) | 103 (11.7) | 53 (1.6) |  |
| Bangladeshi | 11 (0.1) | 29 (0.4) | 91 (5.9) | 23 (2.3) | --- | 54 (6.1) | 10 (0.3) |  |
| Chinese | 17 (0.2) | 18 (0.2) | 59 (3.8) | --- | --- | 7 (0.8) | 14 (0.4) |  |
| Other | 139 (1.6) | 141 (1.9) | 358 (23.3) | 37 (3.7) | 4 (0.5) | 163 (18.5) | 99 (3.1) |  |
| **PLEs** |  |  |  |  |  |  |  |  |
| PSQ 3+ | 27 (0.3) | 33 (0.5) | 18 (1.2) | 19 (2.0) | 23 (3.0) | 48 (5.7) | 52 (1.7) | <0.001 |
| Hypomania | 48 (0.6) | 22 (0.6) | 23 (1.5) | 32 (3.3) | 17 (2.2) | 69 (8.0) | 27 (2.1) | <0.001 |
| Thought insertion | 97 (1.1) | 77 (1.0) | 28 (1.9) | 22 (2.3) | 20 (2.6) | 67 (7.8) | 69 (2.2) | <0.001 |
| Paranoid ideation | 146 (1.7) | 125 (1.7) | 62 (4.2) | 49 (5.1) | 51 (6.6) | 75 (8.7) | 133 (4.3) | <0.001 |
| Strange experiences | 284 (3.3) | 239 (3.2) | 81 (5.5) | 75 (7.8) | 55 (7.2) | 98 (11.4) | 190 (6.1) | <0.001 |
| Hallucinations | 82 (1.0) | 68 (0.9) | 26 (1.8) | 32 (3.4) | 32 (4.2) | 45 (5.3) | 75 (2.4) | <0.001 |

**Supplemental Table 1.** A comparison of the seven population surveys (continued)

|  | Survey 1 | Survey 2 | Survey 3 | Survey 4 | Survey 5 | Survey 6 | Survey 7 |  |
| --- | --- | --- | --- | --- | --- | --- | --- | --- |
|  | n (%) | n (%) | n (%) | n (%) | n (%) | n (%) | n (%) | P^1)^ |
| **Comorbidity** |  |  |  |  |  |  |  |  |
| Depression | 255 (2.97) | 255 (3.4) | 120 (8.1) | 114 (11.8) | 74 (9.5) | 214 (25.3) | 258 (8.2) | <0.001 |
| Anxiety disorder | 1,408 (16.4) | 1,178 (15.9) | 185 (12.4) | 150 (15.6) | 118 (15.2) | 230 (26.8) | 357 (11.4) | <0.001 |
| Alcohol dependence | 567 (6.6) | 367 (5.0) | 69 (4.7) | 100 (10.5) | 93 (12.1) | 186 (22.9) | 222 (7.1) | <0.001 |
| Drug dependence | 258 (3.0) | 200 (2.7) | 11 (0.8) | 22 (2.3) | 53 (6.9) | 63 (7.8) | 52 (1.7) | <0.001 |
| ASPD | 312 (3.7) | 164 (2.2) | 104 (7.1) | 148 (16.1) | 142 (18.4) | 105 (14.0) | 364 (11.9) | <0.001 |
| **Violent behavior** |  |  |  |  |  |  |  |  |
| Any violence | 804 (9.6) | 458 (6.2) | 248 (16.7) | 278 (29.1) | 271 (34.7) | 208 (24.5) | 834 (26.7) | <0.001 |
| Repetitive violence | 184 (2.2) | 64 (0.9) | 29 (2.0) | 49 (5.2) | 50 (6.7) | 58 (6.9) | 123 (4.0) | <0.001 |
| When intoxicated | 337 (4.0) | 201 (2.7) | 74 (5.0) | 126 (13.4) | 183 (23.8) | 105 (12.6) | 421 (13.6) | <0.001 |
| **Severity** |  |  |  |  |  |  |  |  |
| Victim versatility | 40 (0.5) | 51 (0.7) | 22 (1.5) | 36 (3.8) | 53 (6.8) | 23 (2.7) | 101 (3.2) | <0.001 |
| Victim injured | 262 (3.1) | 127 (1.7) | 113 (7.6) | 134 (13.9) | 162 (20.7) | 62 (7.3) | 397 (12.7) | <0.001 |
| Perpetrator injured | 263 (3.1) | 159 (2.2) | 91 (6.1) | 122 (12.7) | 147 (18.8) | 121 (14.2) | 359 (11.5) | <0.001 |
| Police involved | 229 (3.9) | 172 (2.3) | 75 (5.0) | 68 (7.1) | 67 (8.6) | 53 (6.2) | 226 (7.2) | <0.001 |
| Minor violence | 329 (3.9) | 172 (2.3) | 75 (5.0) | 68 (7.1) | 67 (8.6) | 53 (6.2) | 226 (7.2) | <0.001 |
| **Victims of violence** |  |  |  |  |  |  |  |  |
| Intimate partner | 158 (1.9) | 105 (1.4) | 25 (1.7) | 33 (3.4) | 31 (4.0) | 68 (8.0) | 100 (3.2) | <0.001 |
| Family member | 51 (0.6) | 70 (1.0) | 28 (1.9) | 40 (4.2) | 54 (6.9) | 37 (4.3) | 104 (3.3) | <0.001 |
| Friend | 133 (1.6) | 84 (1.1) | 68 (4.6) | 66 (6.9) | 108 (13.8) | 71 (8.3) | 201 (6.4) | <0.001 |
| Person known | 241 (2.9) | 133 (1.8) | 69 (4.6) | 101 (10.5) | 131 (16.8) | 27 (3.2) | 284 (9.1) | <0.001 |
| Stranger | 365 (4.4) | 211 (2.9) | 105 (7.0) | 149 (15.5) | 122 (15.6) | 70 (8.2) | 454 (14.5) | <0.001 |

**Note.** Survey 1: 2000 household survey of Great Britain; Survey 2: 2007 household survey of England; Survey 3: black and ethnic minority men boost sample; Survey 4: men from low socio-economic background boost sample; Survey 5: men from Glasgow East; Survey 6: men from Hackney, London; Survey 7: second Men’s Modern Lifestyle Survey. ^1)^ Based on Pearson chi-square test.
